# Response to comment on: hydrophilic inflatable penile prosthesis surface coatings readily rebind antibiotics and maintain antimicrobial efficacy ex vivo

**DOI:** 10.1038/s41443-025-01220-5

**Published:** 2025-12-08

**Authors:** Brian H. Im, Paul H. Chung

**Affiliations:** Department of Urology, Sidney Kimmel Medical College, Thomas Jefferson University, Philadelphia, PA USA

**Keywords:** Drug delivery, Inflammation

We thank the authors for their kind comments [1]. Given the notably higher rates of infection in penile prosthesis revision surgery (1—3 vs 10%), our aim with this study was to assess the integrity of the hydrophilic surface and its ability to bind and elute antimicrobial solutions for subsequent applications beyond initial implantation [2–4]. We utilized implant reservoirs and cylinders removed from patients, which went through a rigorous sterilization process, for the various aspects of this study. Congo Red staining and contact angle analysis was conducted to assess the integrity of the hydrophilic surface following the sterilization process. Fluorescent-labelled vancomycin was used to directly visualize antibiotic binding to the surface, while antibiotic-dip bacterial assays were conducted to assess the antibacterial efficacy of the previously-implanted surface. This study demonstrates that the surface coating of the pre-existing device has potential to have antibiotics rebind and elute in situ, which may have significant clinical applications; for instance, in cases of component repositioning, as mentioned in the comment by Chawareb et al., the in situ components may potentially re-bind antibiotics via irrigation, rendering the retained components safer [1, 5].

The adaptability and flexibility of the hydrophilic surface, both in primary surgery and subsequent revision cases, is promising, and our understanding of the hydrophilic surface is ever-expanding. Further studies are necessary to maximize the potential of the hydrophilic surface, including optimal concentrations and combinations of antimicrobial solutions (anti-bacterial and anti-fungal), both for primary and revision cases. Additionally, clinical data is necessary to corroborate the findings of our in vitro studies, both to better account for the complex environment in vivo and potential pre-existing biofilms, and to more accurately reflect patient outcomes, as mentioned by the authors of the response. Our knowledge and experience with penile implant infections is evolving as we continue to aim to reduce infection rates and improve outcomes.
